# Dawn of a new day for mental health systems

**DOI:** 10.1186/s13033-021-00475-x

**Published:** 2021-05-26

**Authors:** Daniel Vigo

**Affiliations:** Mental Health Systems and Services Laboratory, Department of Psychiatry and School of Population and Public Health, Faculty of Medicine, University of British Columbia, Vancouver, Canada

In their inaugural Editorial for this Journal, Profs. Harry Minas and Alex Cohen developed a compelling case for why a scientific journal of the highest standards and the broadest possible circulation was sorely needed. Their detailed description of the scope of this Journal’s focus and the daunting gap in knowledge remains as valid today as it was back then, with the exception of the solid body of work that researchers and practitioners from all regions of the world have published in the now well-established International Journal of Mental Health Systems [1].

My first act as Editor-in-Chief is to thank Professor Harry Minas and the Editorial Board for their unique and lasting contribution to science and to the wellbeing of people with mental health-related problems and disorders. And my second act is a call to action to my fellow researchers and practitioners globally: our work has only but started, the impact of our collective efforts as reflected in this Journal has only recently begun to be properly acknowledged. We are at the gates of an unfamiliar world, where mental health has captured the attention of anyone who has been listening to the public conversation about societal priorities. Grass-roots advocates, people with lived experience, their families, public figures in all walks of life—from artists to sports people—have come out through traditional and new media platforms to publicly acknowledge their own mental health struggles and demand adequate funding for mental health research and services. During the past decade, leaders of private and public organizations, jurisdictions, nations, and multilaterals have pledged their commitments to universal health coverage inclusive of mental health, and there is a growing recognition that, in order to achieve this, all levels of research need to advance in lockstep, including basic, clinical, and health systems research. In order to bridge the “knowledge to action” gap (also labeled “bench to bedside” when it comes to health), translational research and implementation science have emerged as a key final step in bringing the advances of our field to the populations in need. It has been found that only 15% of proven innovations ever make it to day-to-day practice, and only after 20 years. This is no surprise when the third crucial step of the translational process (from basic, to clinical, to health systems research) is frequently neglected.

Fourteen years after the IJMHS was created to contribute with evidence to the tide of mental health systems research and practice, where do we stand? I submit that we are at a tipping point: three convergent forces have dramatically changed the landscape and the opportunities at the policy and systems level. First, the global push for universal health coverage inclusive of mental health—driven by the United Nation’s Sustainable Development Goals—has thrust the field into the limelight and significantly expanded the potential sources of funding for mental health systems research. Second, the exponential growth of digital innovation (including data science and online platforms) offers concrete opportunities to effectively deliver services at any scale, highlighting the need for a comprehensive health system strategy capable of integrating e-mental health with in-person services locally, nationally, and globally. And third, the COVID-19 pandemic has painfully demonstrated that even efficacious interventions will only go so far without rational, strategic, system-wide planning capable of delivering them at scale.

## Global commitment to universal health coverage inclusive of mental health

The Sustainable Development Goal number 3 (specifically targets 3.4, 3.5, and 3.8) embodies an explicit pledge by all Governments to implement the required changes at the national level to meet its targets. In addition to committing national research funds to mental health research, this pledge gives multilateral organizations the mandate to facilitate these changes by developing innovative financing mechanisms to support scaleup and evaluation. Indeed, the United Nations and its agencies (most notably the World Health Organization (WHO), but also all others) now have legitimacy to prioritize mental health as one of the central health and development challenges of our time, as they have successfully done in the past with HIV and maternal and child health. The World Bank and other agencies are incentivized to include mental health components in development assistance for health packages, as well as mental health impact assessments in general development packages (analogous to environmental impact assessments for infrastructure projects). Also, debt-for-mental-health swaps can now be considered by high-income creditors for debt-distressed countries, swapping payments for capital and/or interest for local mental health investments. When it comes to global development priorities, mental health is poised to take the central stage previously occupied by the fight against AIDS and the push to improve maternal and child health outcomes.

## Digital mental health

Until recently, calls to scale up mental health services—justified as they may be in light of the unparalleled mental health burdens and the cost–benefit evidence—lacked a feasible, credible plan. The shortage and high cost of human resources for mental health has been the hill where most policy battles ended, certainly in low-income, but also in high-income countries. Two critical innovations, one low-tech and one high-tech, have transformed the landscape: the solid evidence supporting task-shifting (also referred to as task-sharing or right-skilling, depending on the conceptual framework), combined with the infinitely scalable nature of digital tools, offer a concrete program of global scale-up with minimal marginal cost. This combination allows for a rapid expansion of coverage leveraging the ubiquitous distribution of smartphones in middle- and high-income settings. For low-income settings, additional efforts are required to distribute devices, create digital hubs, and expand network coverage to deliver self-guided and peer-supported tools, as well as clinical training, supervision, and services. Task shifting and leveraging digital tools represent quintessential health systems innovations: they offer platforms to deliver interventions that have already been found to be clinically effective, from psychotropics to cognitive behavioural therapy (CBT), peer-support and self-care, but were until now largely undeliverable due to the prohibitive cost of the specialized brick-and-mortar and personnel required.

## The importance of a health systems perspective

The first wave of the Covid-19 pandemic has driven home the message of how costly it is to have unprepared, fragmented systems. Having well-functioning, integrated systems able to gather data about population needs and to respond in a coordinated manner helps explain why some countries withstood the first wave relatively unscathed with respect to health outcomes, and why vaccination rollout efforts met with different degrees of success, even in similar country-income levels. For our field, the implications loom as large, if less noticeable: the evidence showing that online psychotherapy, peer-supports, and self-guided care work is already out there; what is lacking are the pragmatic, real-world implementation and translational studies assessing the impact of integrating these innovations into existing health systems. These types of program evaluations, most of them grounded on mixed-methods and/or multi-level analyses considering not only individual effectiveness but also the organizational or system level impacts are what the mental health field needs at this point to quantify and qualify what is gained—and what may be at risk of being lost—by this unprecedented democratization of mental health services.

In summary, the IJMHS seeks to foster the critical research programs that will inform the decision-making process required to democratize mental health services, by bringing together the multiple effective interventions we already know to exist and the platforms that will deliver them at scale in real-world health systems. In order to achieve these goals, we are adding four Editorial Priority Areas to the general focus of our journal, each with a new responsible Associate Editor. *Digital Heath and Mental Health Systems* will seek to incentivize and disseminate research focusing on the integration of digital tools (from virtual care and telemedicine to fully automated AI-powered interventions) into existing systems and services. The *Substance Use and Mental Health Systems* area will highlight the need to consider mental health and substance use together when developing services and systems, in a world where the patterns of substance use are rapidly shifting (from the opioid crisis in its multiple forms to the various decriminalization, legalization, and normalization efforts). The *Mental Health Systems Planning* Editorial Priority Area will encourage research and submissions that stem from collaborations between mental health researchers and decision-makers geared towards informing prioritization and resource allocation by rigorously assessing what the population needs are and what the services to meet those needs should be, both from a qualitative and a quantitative perspective. And finally, *Mental Health Systems for Populations in Need*: this Editorial Priority Area will focus on population groups that are particularly underserved from a mental health systems perspective. These groups may be underserved due to different reasons: they may constitute a group historically neglected by our field, as is the case with older adults; or a group whose needs are prioritized but poorly understood, such as children, adolescents, and transitional age youth; or a historically marginalized group, such as indigenous peoples everywhere.

This is an ambitious program of editorial work that seeks to build on the achievements made possible by Harry Minas, the Editorial team, and mental health systems researchers everywhere. Of note, IJMHS will continue to be a reliable source not only for studies from high-income settings, but also for knowledge and innovations from low- and middle-income countries. The two examples of health systems innovation highlighted above demonstrate how scientific advances are indeed bidirectional: computer science and digital tools emerged in the wealthiest countries, as innovators sought to maximize our ability to gather, store, access, curate, analyze, and deliver data; conversely, task-shifting emerged in low-income settings, as innovators strove to meet people’s needs despite lacking the amount and types of resources (both material and human) that were traditionally considered necessary. One limitation of most international journals is their insufficient focus on knowledge emerging from low- and middle-income countries (LAMICS). This is sometimes due to the dominance of the English language, the rigidity of editorial expectations, or the chronic underfunding of research in LAMICS. It may also be the result of less explicit but more deeply rooted biases stemming from the colonial history of our discipline and science in general. With this in mind, and building on the track record of our journal, we will create a new section, *Field Experiences*, inspired by the World Health Organization Bulletin’s Lessons from the Field, with the goal of facilitating the publication of unique health systems experiences that for different reasons may lend themselves more to a “case study” format than to traditional research papers. We will also seek to strengthen our collaboration with regional research networks to create a pipeline that increases the visibility of the vast mental health systems knowledge currently being produced in LAMICS.

We hope you find this Editorial proposition engaging, and that you are inspired to submit your research to our journal and even to develop projects or programs targeting the areas mentioned above. This is the right time to focus your research funding proposals on mental health systems.
